
JOURNAL INFORMATION
==============================
NLM Title Abbreviation: Behav Neurol
Journal ID: Behav Neurol
NLM Journal ID: 8914585
Journal ID: BN

Behavioural Neurology

ISSN: 0953-4180
EISSN: 1875-8584
Publisher: Wiley

ARTICLE INFORMATION
==============================
PMCID: PMC5215105
PMID: 22713413
DOI: 10.3233/BEN-2012-120272
Article ID: 473642
Article version: 1
Subjects: Editorial

The Behavioural Neurology of Basal Ganglia Disorders

Cavanna Andrea Eugenio 1 2 *
1 The Michael Trimble Neuropsychiatry Research Group Department of Neuropsychiatry BSMHFT and University of Birmingham Birmingham UK
2 Department of Motor Neuroscience and Movement Disorders Institute of Neurology and University College London London UK
*Andrea Eugenio Cavanna: a.cavanna@ion.ucl.ac.uk
Print publication date: 2013
Volume: 26
Issue: 4
First page: 217
Last page: 218
Received 2012 May 22; Accepted 2012 May 22
Copyright: Copyright © 2013 Hindawi Publishing Corporation and the authors.
Copyright year: 2013
License: This is an open access article distributed under the Creative Commons Attribution License, which permits unrestricted use, distribution, and reproduction in any medium, provided the original work is properly cited.
License URL: https://creativecommons.org/licenses/by/3.0/

==============================
Behavioural Neurology 26 (2013) 217–218
DOI 10.3233/BEN-2012-120272
IOS Press

Editorial

### The behavioural neurology of basal ganglia disorders

Andrea Eugenio Cavanna[a,b,*]
[a]*The Michael Trimble Neuropsychiatry Research Group, Department of Neuropsychiatry, BSMHFT and University of Birmingham, Birmingham, UK*
[b]*Department of Motor Neuroscience and Movement Disorders, Institute of Neurology and University College London, London, UK*

Basal ganglia disorders are common clinical conditions characterised by specific motor dysfunction and associated behavioral problems. The underlying pathophysiology primarily consists in alterations of the cortico-striato-thalamo-cortical pathways, which can be related to both neurodegenerative pathology (e.g. Parkinson disease, Huntington disease) and neurodevelopmental processes (e.g. Tourette syndrome, dystonia). Modulation of dopaminergic neurotransmission within the basal ganglia circuitries represents the mainstay of pharmacological treatment for these conditions. This thematic issue on "The behavioural neurology of basal ganglia disorders" explores the pathophysiological mechanisms underlying the most common basal ganglia disorders, with special focus on the neurochemical targets for pharmacotherapy.

The introductory article (Ward et al.) presents the functional neuroanatomy and behavioral correlates of basal ganglia as demonstrated by case studies of patients with selective lesions affecting the cortico-basal pathways. Lesions involving the caudate nucleus, putamen and globus pallidus have been found to be associated with a spectrum of behavioral and cognitive symptoms, including abulia, poor working memory and deficits in emotional recognition. The paper by Alobaidi and Pall focuses on the pharmacotherapy of Parkinson disease, an area which is often challenging as clinicians have to find a delicate balance between the efficacy on motor symptoms and the side effects profiles of different dopaminergic medications aimed at restoring basal ganglia activity. This article assesses the available evidence on the role of dopamine agonist monotherapy as an alternative to Levodopa in the treatment of the motor symptoms of Parkinson disease, along with the role of dopamine antagonists in the treatment of Parkinson disease -related psychosis. A related area of high interest over the last few years has been the pathophysiology of impulse control disorders in patients with Parkinson disease: this topic is covered by the following article (Balarajah and Cavanna), along with the implications of these behavioral symptoms for the pharmacological management of Parkinson disease. Another neurodegenerative disorder at the interface between neurology and psychiatry is Huntington disease. The neuropathological process of this condition also involves the basal ganglia, and has been solidly anchored to genetic bases. The paper by Pidgeon and Rickards covers the current evidence supporting the pharmacological treatment for both motor and non-motor symptoms of Huntington disease, whilst Mittal and Eddy's contribution focuses on the pharmacological modulation of dopaminergic and glutamatergic pathways, which are crucially affected in this condition. The article by Waldon et

*Correspondence: Dr. Andrea Eugenio Cavanna MD, Department of Neuropsychiatry, The Barberry National Centre for Mental Health, Birmingham B15 2FG, UK. Tel.: +44 121 3012317; Fax: +44 121 3012291; E-mail: a.cavanna@ion.ucl.ac.uk.

ISSN 0953-4180/13/$27.50 © 2013 – IOS Press and the authors. All rights reserved al. offers an overview of the evidence-based treatment of tic symptoms in Tourette syndrome, a quintessentially neuropsychiatric basal ganglia disorder, in which challenging behavioural problems often accompany the motor and vocal manifestations. The paper by Patel and Martino presents a systematic review on the evidence-based pharmacotherapy for cervical dystonia, whilst the closing article by Jones and Cavanna focuses on the pathophysiology and treatment of a common and often under-recognized condition, restless legs syndrome.

It is the editor's hope that this timely issue on basal ganglia disorders will prove to be a useful resource for both basic and clinical researchers, plus neuropsychiatrists with a special interest in movement disorders. Finally, this issue is proposed as an up-to-date reference for clinicians opting for the pharmacological modulation of what Ivan Pavlov intuitively called "the blind force of the subcortex".
